# Turkish urologists’ knowledge and attitudes on lifestyle advice for bladder cancer: a national survey

**DOI:** 10.1007/s00520-026-10496-2

**Published:** 2026-02-28

**Authors:** Reha Girgin, Necmettin Aydın Mungan

**Affiliations:** Faculty of Medicine, Department of Urology, Zonguldak Bulent Ecevit University, Zonguldak, Turkey

**Keywords:** Bladder cancer, Non-muscle invasive bladder cancer, Urologists, Life style, Surveys and questionnaires

## Abstract

**Purpose:**

To evaluate the knowledge level of Turkish urologists regarding the lifestyle changes related to bladder cancer and to what extent they question patients’ lifestyles and guide them in making recommended changes.

**Methods:**

A 14-question online survey, based on a questionnaire prepared by Beeren et al. (Bladder Cancer 10(3):215-220, 2024), was sent to Turkish urologists. The survey included demographics, familiarity with guidelines, lifestyle assessment and advice, and perceived barriers during care for BC patients.

**Results:**

The mean age of the 252 participants was (44.8 ± 9.3), approximately 40% were affiliated with an academic hospital, and approximately 50% had more than 10 years of experience. Almost all were interested in uro-oncology, with 30% devoting more than half of their daily practice to uro-oncology. Smoking was reported as the most frequently questioned issue, for which advice was given and referrals to a lifestyle specialist provided when necessary. Asking about and giving advice regarding fluid intake came second, with approximately 50% mentioning this issue. Referral rates were low for lifestyle factors other than smoking. The most frequently reported barriers were a lack of information about where patients should be referred, insufficient motivation of patients, and concern about blaming patients.

There is a positive correlation between knowledge on ideal body weight and physical activity with asking about lifestyle changes (*p* = 0.004, *p* = 0.000), and giving lifestyle advice (*p* = 0.014, *p* = 0.003) and between knowledge on ideal body weight with referring for lifestyle advice and perception in lifestyle factors (*p* = 0.035, *p* = 0.016).

**Conclusions:**

Lifestyle changes in bladder cancer patients are not sufficiently integrated into routine clinical practice by urologists. Although lifestyle changes are perceived as important by most urologists, they encounter some barriers in making these recommendations to their patients.

## Introduction

Bladder cancer (BC) is the tenth most common cancer, and 75% of cases are non-muscle invasive bladder cancer (NMIBC) at the time of initial diagnosis [1]. Although NMIBC has promising 5-year survival rates, there are varying recurrence and progression rates depending on the disease characteristics [1]. This necessitates costly, invasive, and sometimes challenging follow-up programmes for patients.

The relationship between patients’ habits and some types of cancer is known [2]. The World Cancer Research Fund/American Institute for Cancer Research (WCRF/AICR) recommendations in 2018 included maintaining an ideal weight, eating a healthy diet, engaging in physical activity, and reducing alcohol consumption [3]. Studies have shown that a cancer diagnosis significantly increases one’s compliance with lifestyle changes [4, 5]. In this regard, compliance is important in general, not just in one area, to achieve the desired result [6]. Smoking, environmental pollutants, obesity, metabolic syndrome, sedentary lifestyle, and excessive alcohol use have been shown to play a role in the development and recurrence of bladder cancer [7–12].

Patients in general are often highly motivated to change their lifestyle behaviour when they first receive a cancer diagnosis [13]. BC patients have also been shown to be more likely to accept smoking as a risk factor when their urologists emphasise it at the initial diagnosis [14].

However, studies have shown that compliance with multiple lifestyle changes is low in BC patients in the long term [15]. This highlights the importance of physicians discussing lifestyle changes with their patients.

Due to possible gaps in local clinical guidelines, we aim to determine the extent to which Turkish urologists question, recommend, or make referrals regarding lifestyle changes to patients with BC. Our secondary aim was to assess the extent the barriers Turkish urologists encounter in providing recommendations.

## Methods

To examine the lifestyle recommendations in the treatment management of BC patients, an online survey was created based on a questionnaire developed by Beeren et al. [16]. After the linguistic translation of the original questionnaire, its content validity and applicability were assessed by two independent urologists with at least 10 years of experience in the field of uro-oncology. The final version of the questionnaire was developed based on their suggestions.

The participants’ ages, work units, work experiences, academic title, and areas of expertise were examined. In addition to the participants’ mastery of the guidelines, regarding lifestyle recommendations were assessed using multiple-choice answers. The extent to which they ask questions, give advice, and refer people for lifestyle coaching was assessed using a 5-point Likert scale that represents the frequency with which these practices occur in general. (Scaled as no one/< 25%/26–50%/51–75%/75% <). The perceptions of the participants were assessed on a 4-point Likert scale: very unimportant, unimportant, important, and very important.

The survey consisted of 14 questions and, finally, a question inquiring about their personal opinions and the average response time was 2 min. To ensure that the participants completed the questionnaire only once, the design was such that it could only be filled out through an internet protocol.

The collection of survey responses began after ethical approval was obtained (Zonguldak Bulent Ecevit University non-interventional clinic research ethics committee date: 04/12/2024, no: 2024/21). Google Docs™ was used to prepare the survey form, as it ensured the participants’ anonymity and was sent to doctors registered in the Society of Urological Surgery (UCD) database working in Turkey via phone application (WhatsApp groups) and email between January and February 2025. The survey was conducted in accordance with the “Declaration of Helsinki” and after providing online informed consent from the participants. The responses received at the end of the period were analysed and evaluated.

All analyses were performed using the IBM (Armonk, NY, USA) SPSS Statistics Version 22 statistical software package. Categorical variables were expressed as numbers and percentages. A multinomial logistic regression model was performed to evaluate degree of influence of familiarity with lifestyle modification guidelines and the time devoted to uro-oncology in daily practice.

This provided us with the opportunity to examine the relationship between guideline awareness as a dependent variable and the participants’ frequency of involvement in the field of uro-oncology as an independent variable. Statistical significance for the coefficients was achieved with a *p*-value < 0.05, indicating whether the attribute has an effect on awareness. The normal distribution of numerical variables was assessed using the Shapiro–Wilk test. The Kruskal–Wallis test was used to compare the areas of interest in the field of uro-oncology with familiarity with guidelines, questioning, giving advice, and perceiving the importance of lifestyle changes, while Spearman’s test was used to calculate the correlation coefficient (rho) between them. The statistical significance level was accepted as *p*-value < 0.05.

## Results

**Fig. 1:**
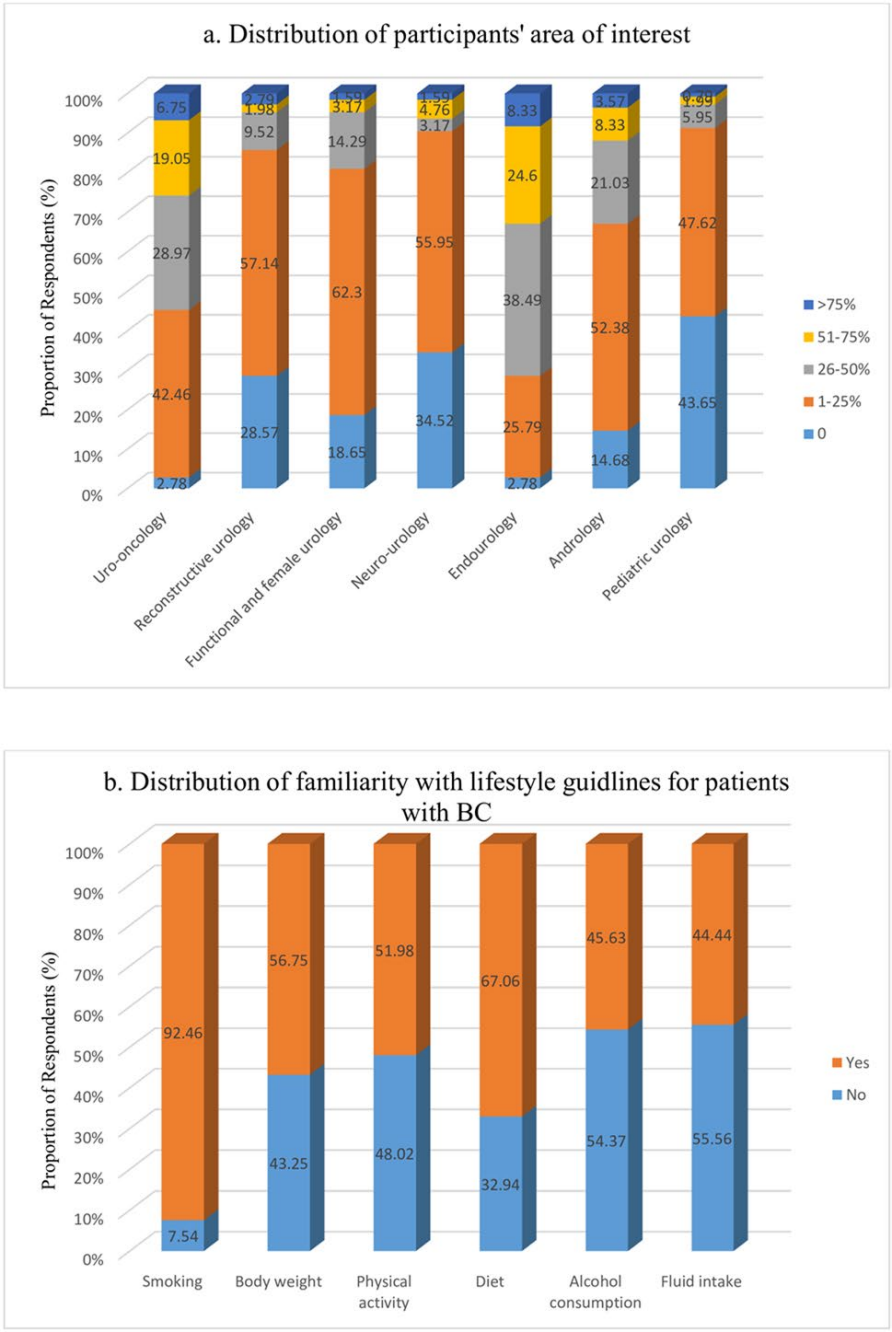
**a** Distribution of participants’ area of interest. **b** Distribution of familiarity with lifestyle guidlines for patients with BC

Of the 252/1300 (20.16%) respondents who completed the questionnaire, 99.6% (251) were male with a mean age of (44.8 ± 9.3). Approximately one-quarter of the participants (25.8%) stated that they devoted more than half of their time to uro-oncological cases. A summary of the areas of interest of participants is shown in Fig. 1a. Twenty-five (9.9%) participants were freelancers, 58 (23%) worked in a private hospital, 56 (22.2%) worked in a university hospital, and the rest worked in different state hospitals. Ninety-eight (38.9%) participants worked as specialists, while the rest worked at different academic levels. Ninety (35.7%) participants had 11–20 years of experience, while 69 (27.4%) had more than 20 years of experience. The participants’ demographic data are summarised in Table 1.

**Table 1:** Demographic features

**Working center**	*n*	%
State hospital	31	12.3
Private hospital	58	23.0
City hospital	32	12.7
Medical faculty hospital	56	22.2
Education research	50	19.8
Free	25	9.9
**Title**		
Specialist Dr	98	38.9
Assistant Prof	22	8.7
Assoc. Prof	69	27.4
Prof. Dr	63	25.0
**Urology experience (years)**		
0–1	13	5.2
2–5	40	15.9
6–10	40	15.9
11–20	90	35.7
> 20	69	27.4

Of the participants, 233 (92.5%) stated that they were familiar with at least one guideline. Fifty-three (21.0%) reported that they were familiar with each guideline. Familiarity with the guidelines on smoking was highest, at 92.5%. Familiarity with the guidelines is summarised in Fig. 1b.

**Fig. 2:**
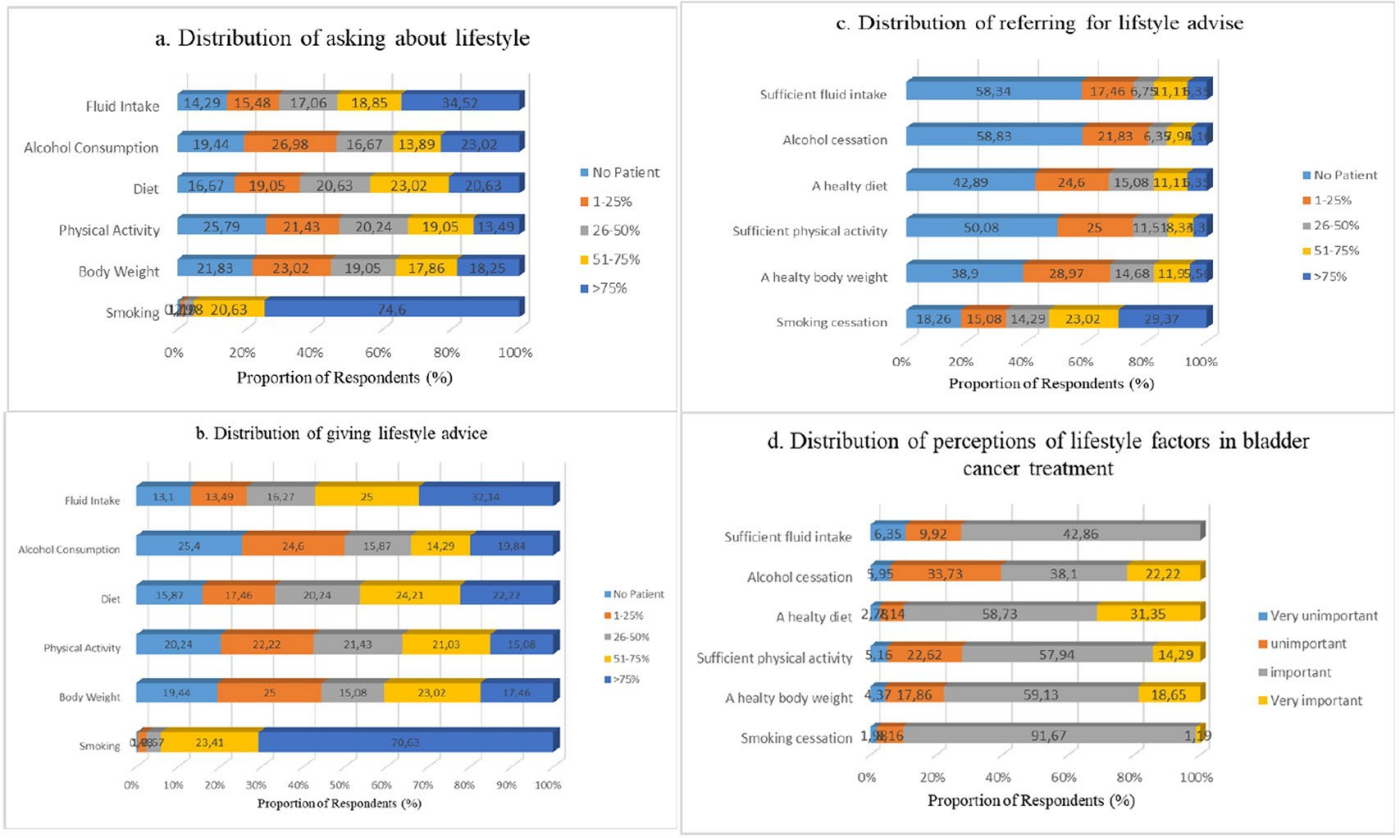
**a** Distribution of asking about lifestyle. **b **Distribution of giving lifestyle advice. **c **Distribution of referringfor lifestyle advice. **d** Distribution of perceptions of lifestyle factors in bladder cancer treatment

It was observed that the participants questioned their patients frequently about their smoking habits and advised them to cease smoking. Regarding other lifestyle changes besides smoking, 14.29–25.79% did not ask any questions, and 13.1–25.4% did not make any recommendations (Fig. 2a and b).

Only one-third of participants advised more than 75% of their patients to cessate smoking. Almost half of the participants did not refer their patients for help with other lifestyle changes (Fig. 2c).

Most participants indicated that stopping smoking was important, followed by eating a healthy diet and drinking enough fluids. However, while the rate of those who think that “consuming enough fluids” is very important was 40%, the rate of those who consider “cessation of smoking” to be very important was only 1.19%. The proportion of those who thought that other lifestyle changes were not important varied between 22.23% and 39.68% (Fig. 2d).

**Fig. 3:**
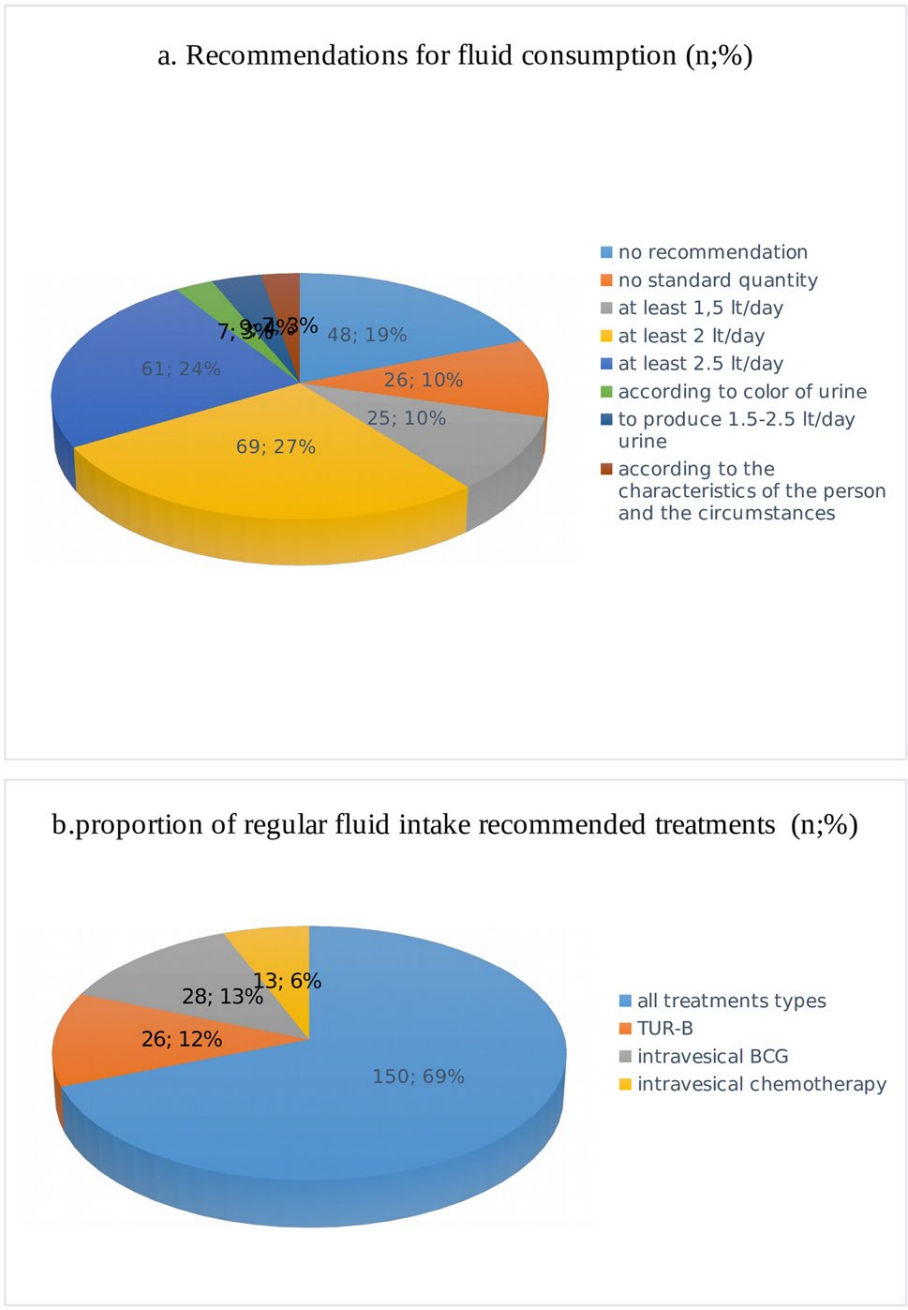
**a** Recommendations for fluid consumption (*n*; %). **b **Proportion of regular fluid intake recommended treatments (*n*;%)

Nineteen per cent of the participants reported that they did not make recommendations to their patients regarding fluid intake, and about 10% did not provide an opinion on a standard intake amount. Approximately 60% of the remaining participants recommended 1.5 to 2.5 L of fluid intake per day, while 10% reported that they made their recommendations based on personal, environmental, and urinary characteristics (Fig. 3a). Approximately 70% of the urologists reported that they provided advice about increasing fluid intake to all treatment types for NMIBC, while the remaining 30% reported that they only emphasised this during transurethral resection of the bladder (TURB), intravesical Bacillus Calmette-Guerin (BCG), and chemotherapy applications (Fig. 3b).

No statistically significant relationship was observed between the time participants devoted to uro-oncology and their knowledge of lifestyle guidelines. Although the results did not show a statistical significance, the odds ratios were very low and the confidence intervals were very wide, which may be related to the small sample size (Table 2).

**Table 2:** The relationship between urologists’ awareness of the guidelines and their frequency of involvement in uro-oncology in daily practice

Participants’ interest in the field of uro-oncology (*n* = 252)						
Ref = none	Smoking OR (95% CI)	Body weight OR (95% CI)	Physical activity OR (95% CI)	Diet OR (95% CI)	Alcohol consumption OR (95% CI)	Fluid intake OR (95% CI)
**1–25%**	0.02 (0.00–0.79)*	0.23 (0.02–3.21)	0.67 (0.04–10.32)	0.11 (0.01–1.50)	2.73 (0.32–23.37)	0.59 (0.05–6.32)
**26–50%**	0.07 (0.00–2.40)	0.18 (0.01–2.55)	0.54 (0.04–8.54)	0.13 (0.01–1.86)	4.13 (0.47–36.37)	0.63 (0.06–6.91)
**51–75%**	0.02 (0.00–1.32)	0.09 (0.01–1.38)	0.33 (0.02–5.45)	0.12 (0.01–1.81)	5.16 (0.55–48.32)	0.62 (0.05–7.16)
** > 75%**	0.02 (0.00–1.32)	0.09 (0.05–1.63)	0.28 (0.01–5.42)	0.29 (0.02–5.2)	2.77 (0.25–30.30)	0.52 (0.04–6.87)

A multinomial logistic regression model with awareness of lifestyle guidelines (Y/N) and interest in the field of uro-oncology (none/1–25%/26–50%/51–75%/> 75%) as the dependent variable

Ref: Having no idea

*OR*, odds ratio; *CI*, confidence intervals

^*^p < 0.05

When the correlation between lifestyle guidelines and the approach to lifestyle change among urologists interested in the field of uro-oncology was examined, there is a positive relationship between the topic of ideal body weight and physical activity with asking about lifestyle (*p* = 0.004, *p* = 0.000), and giving lifestyle advise (*p* = 0.014, *p *= 0.003) and between the topic of ideal body weight with referring for lifestyle advise and perception in lifestyle factors (*p *= 0.035, *p* = 0.016) (Table 3).

**Table 3:** The correlation between lifestyle guidelines and the approach to lifestyle change among urologists interested in the field of uro-oncology

		In the field of uro-oncology					
		Smoking	Body weight	Physical activity	Diet	Alcohol consumtion	Fluid intake
Asking about lifestyle^π^	*r*	0.061	0.183	0.226	0.014	0.116	0.033
	*p*	0.332	0.004*	0.000*	0.830	0.067	0.598
Giving lifestyle advice^π^	*r*	−0.028	0.155	0.189	0.086	0.120	0.085
	*p*	0.654	0.014*	0.003*	0.172	0.058	0.181
Referring for lifestyle advice^π^	*r*	0.110	0.133	0.101	0.055	0.026	0.029
	*p*	0.082	0.035*	0.110	0.388	0.681	0.648
Perception in lifestyle factors^π^	*r*	−0.023	0.151	0.122	0.033	−0.001	0.022
	*p*	0.718	0.016*	0.052	0.603	0.992	0.731

*π*, Kruskal-Wallis test; *r*, Spearman correlation coefficient

^*^p < 0.05

“Lack of interest from patients around lifestyle recommendations” was the most frequently reported barrier, at 69.8%, and “insufficient knowledge of referral options” was reported as the second most frequent barrier, at 61.5%. The barriers and their percentages are summarised in Table 4.

**Table 4:** Barriers experienced by respondents in giving lifestyle recommendations to patients with bladder cancer

Barriers	According to participants’ interest in the field of uro-oncology (*n*, %)					Total response
	**None**	**1–25%**	**26–50%**	**51–75%**	** > 75%**	***n*, %**
Insufficient evidence on the importance of lifestyle recommendations for bladder cancer outcomes	1 (2.1)	20 (42.6)	19 (40.4)	6 (12.8)	1 (2.1)	47 (18.7)
Insufficient knowledge on having an effective conversation about lifestyle	1 (5.0)	11 (55.0)	6 (30.0)	2 (10.0)	0 (0)	20 (7.9)
Insufficient knowledge on referral options	4 (2.6)	62 (40.0)	50 (32.3)	27 (17.4)	12 (7.7)	155 (61.5)
Lack of interest from patient in lifestyle recommendations	6 (3.4)	72 (40.9)	50 (28.4)	36 (20.5)	12 (6.8)	176 (69.8)
Insufficient motivation from patient to follow given lifestyle recommendations	2 (3.2)	26 (41.3)	23 (36.5)	7 (11.1)	5 (7.9)	63 (25.0)
Poor overall condition of the patient	0 (0)	5 (31.3)	8 (50.0)	3 (18.8)	0 (0)	16 (6.3)
Possibly creating the impression that the patient is to blame	3 (2.7)	47 (42.3)	33 (29.7)	18 (16.2)	10 (9.0)	111 (44.0)
Lack of time during an outpatient clinic visit	2 (2.9)	22 (31.9)	23 (33.3)	14 (20.3)	8 (11.6)	69(27.4)
Lifestyle advice is not part of the responsibilities of a urologist	0 (0)	1 (16.7)	3 (50.0)	2 (33.3)	0 (0)	6 (2.4)
I do not experience any barriers in giving lifestyle recommendations	0 (0)	26 (52.5)	8 (17.5)	11 (22.5)	3 (7.5)	48 (19.0)

## Discussion

In this study, we evaluated urology specialists with a survey, and it was seen that apart from the issue of smoking, the issue of lifestyle changes was not given sufficient importance in the evaluation of patients with BC.

Although the rates for asking about smoking habits and giving advice about smoking were high, it was observed that the rates of referral to a specialist for smoking cessation were low. However, smoking cessation was considered important among urology specialists. A recent literature review showed studies reporting similar results [16–18].

In our study, it was observed that there was no relationship between the time urologists spent in the field of uro-oncology and their familiarity with smoking-related guidelines. It was also seen that working in the field of uro-oncology did not change the rate of asking questions, giving advice, and taking the issue of lifestyle changes regarding smoking seriously. On the contrary, a study by Matulewicz et al. showed that urologists working in the field of oncology take this issue more seriously [18]. The literature shows that smoking cessation rates in patients diagnosed with bladder cancer are low [16, 19, 20].

Considering that BC patients should be aware of their ideal body weight, engage in regular activity, eat an appropriate diet, reduce alcohol consumption, and drink sufficient fluids as lifestyle changes, the data obtained from our study show that urologists did not score above average in this regard. This finding was consistent with the fact that approximately half of the urologists were not familiar with the guidelines on these issues. In addition, it was seen that working in the field of uro-oncology did not change this pattern. A study conducted by Pallin et al. among radiation oncology specialists showed that the knowledge of the guidelines was around 50% [21]. In a study conducted by Koutoukidis et al. among healthcare professionals, the level of knowledge was similarly low [22]. However, the literature shows that with increasing awareness of the guidelines, questioning patients on their lifestyle habits and giving them lifestyle advice also increases [21, 23]. In addition, the literature shows that patients do not take these lifestyle changes seriously enough [7, 19]. Therefore, it is important to constantly inform and warn patients about the need to adopt lifestyle changes.

Approximately 80% of the participants reported that they encountered barriers, the most common of which were patients’ lack of interest in lifestyle change recommendations and a lack of information about where to refer them. Interestingly, almost half of the urologists hesitated to blame their patients. These rates were higher than those reported in the literature [16, 21]. We believe that this can be explained by differences in patient profiles and conditions. However, we observed that urologists who work intensively in uro-oncology were less affected by these barriers. This suggests that having received training on a specific topic may enable urologists to easily handle a specific patient and disease group. Approximately 25% of urologists felt that the guidelines did not provide enough evidence to justify making lifestyle recommendations or stated that they did not have enough information to make adequate recommendations. Interestingly, although higher rates of familiarity with the guidelines were reported in the studies by Beeren et al. [16] and Pallin et al. [21], higher rates of barriers were also reported. This suggests that simply being familiar with the guidelines may not be sufficient, and that being knowledgeable about lifestyle recommendations for BC patients may need to be addressed differently.

The current healthcare system’s focus on diagnosis and treatment, coupled with factors such as high population density, patient overload, and inadequate primary care infrastructure, limits the time available for each patient. These inadequacies can also lead to various issues, such as quality of life and lifestyle changes, being overlooked in daily clinical practice.

Given the relationship between lifestyle changes and bladder cancer prognosis, it appears that lifestyle modifications should be incorporated into public health programmes. In this context, as our study demonstrates, urologists fail to demonstrate sufficient care in this area for various reasons. Therefore, implementing medical education and in-service training programmes for daily practice would be beneficial not only for public health but also for the national budget.

### Limitations

This study is the first to evaluate the lifestyle change approaches that urologists in Turkey provide to BC patients. It provides us to make a more homogeneous comment, especially when different work units, fields of interest, and experience levels are taken into account. The fact that approximately half of the participants had more than 10 years of professional experience increases the reliability of the data. In addition, approximately half of the participants were at an academic level that indicates their continuous acquisition of knowledge.

However, our results may have been influenced by potential response bias, such as whether urologists more involved in lifestyle medicine were likelier to participate and whether awareness or positive attitudes may have been exaggerated.

Our study focused specifically on urologists who are interested in all fields of urology because we think that being interested only in uro-oncology may negatively affect the evaluation of the approach to this issue. Approximately 25% of the participants reported that they deal with uro-oncology intensively in their daily practice. This provided us with an effective interpretative ability.

One of the weaknesses of our study is that only a small number of urologists from across the country participated. This may not be representative of all urologists across Turkey. A wider participation would have provided more reliable interpretations.

Nevertheless, this study provides a comprehensive overview of current practices among urologists treating bladder cancer patients in Turkey, including key perceptions of and barriers to lifestyle recommendations. Therefore, it may provide direction for future studies on the lifestyles of bladder cancer patients.

## Conclusion

Our study demonstrates that Turkish urologists consider their patients’ lifestyles when managing bladder cancer, but ultimately, this approach falls short. Although urologists largely perceive the importance of lifestyle, we recommend that this weakness in practice should be addressed and the training processes, including local guidelines, around this issue should be re-evaluated. Increasing the amount of lifestyle-related advice provided by urologists and simultaneously increasing the number of patients seeking it is crucial.

## Data Availability

No datasets were generated or analysed during the current study.
